# Emerging polymeric electrospun fibers: From structural diversity to application in flexible bioelectronics and tissue engineering

**DOI:** 10.1002/EXP.20210029

**Published:** 2022-01-28

**Authors:** Xingyi Wan, Yunchao Zhao, Zhou Li, Linlin Li

**Affiliations:** ^1^ Beijing Institute of Nanoenergy and Nanosystems Chinese Academy for Sciences Beijing P. R. China; ^2^ School of Nanoscience and Technology University of Chinese Academy of Sciences Beijing P. R. China; ^3^ Center on Nanoenergy Research School of Physical Science and Technology Guangxi University Nanning P. R. China

**Keywords:** bioelectronics, electrospinning, fiber, scaffold, tissue engineering

## Abstract

Electrospinning (e‐spin) technique has emerged as a versatile and feasible pathway for constructing diverse polymeric fabric structures, which show potential applications in many biological and biomedical fields. Owing to the advantages of adjustable mechanics, designable structures, versatile surface multi‐functionalization, and biomimetic capability to natural tissue, remarkable progress has been made in flexible bioelectronics and tissue engineering for the sensing and therapeutic purposes. In this perspective, we review recent works on design of the hierarchically structured e‐spin fibers, as well as, the fabrication strategies from one‐dimensional individual fiber (1D) to three‐dimensional (3D) fiber arrangements adaptive to specific applications. Then, we focus on the most cutting‐edge progress of their applications in flexible bioelectronics and tissue engineering. Finally, we propose future challenges and perspectives for promoting electrospun fiber‐based products toward industrialized, intelligent, multifunctional, and safe applications.

## Graphical abstract

Electrospinning (e‐spin) technique has emerged as a versatile and feasible pathway for constructing polymeric fabric with structural diversity, which show potential applications in flexible bioelectronics and tissue engineering for the sensing and therapeutic purposes. This perspective focus on the fabrication strategies of hierarchically structured e‐spin fibers as well as the most cutting‐edge progress in these application fields. The future challenges and prospects are also highlighted.

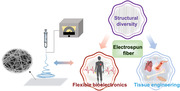

## 1 INTRODUCTION

The existence of fibrous structures in the form of continuous network or individual elongated substance are ubiquitous in the living system, such as, collagenous fibril, muscle fiber, and tendon. These natural structures and organizations inspire the researchers to develop man‐made fibers for biological, biomedical, and other applications. Electrospinning (e‐spin) has been developed as a powerful technique allowing for stable production of fibers with tunable diameters ranging from a few nanometers to several micrometers. E‐spin technology is based on the combinational process of electrostatic interaction and spinning. During the process, the viscoelastic liquid droplet in high electric field is electrified to generate a jet to be ejected, followed by the elongation and acceleration to produce fibers once the electric force of induced charges on the liquid overwhelms the surface tension.^[^ ^1^ ^]^ The fabric structure fabricated from e‐spin can have tissue‐like structural and mechanical properties to simulate natural biological tissues, therefore realizing a bionic manufacturing. Recently, the flourishing development of this technology provides alternatives to manufacture, assembly and processing of the next‐generation materials and devices in numerous fields including air filtration,^[^ ^2^ ^]^ water treatment,^[^ ^3^ ^]^ catalysis,^[^ ^4^ ^]^ food technology,^[^ ^5^ ^]^ photonics,^[^ ^6^ ^]^ flexible electronics,^[^ ^7^ ^]^ and biomedical engineering.^[^ ^8^ ^]^

**FIGURE 1:**
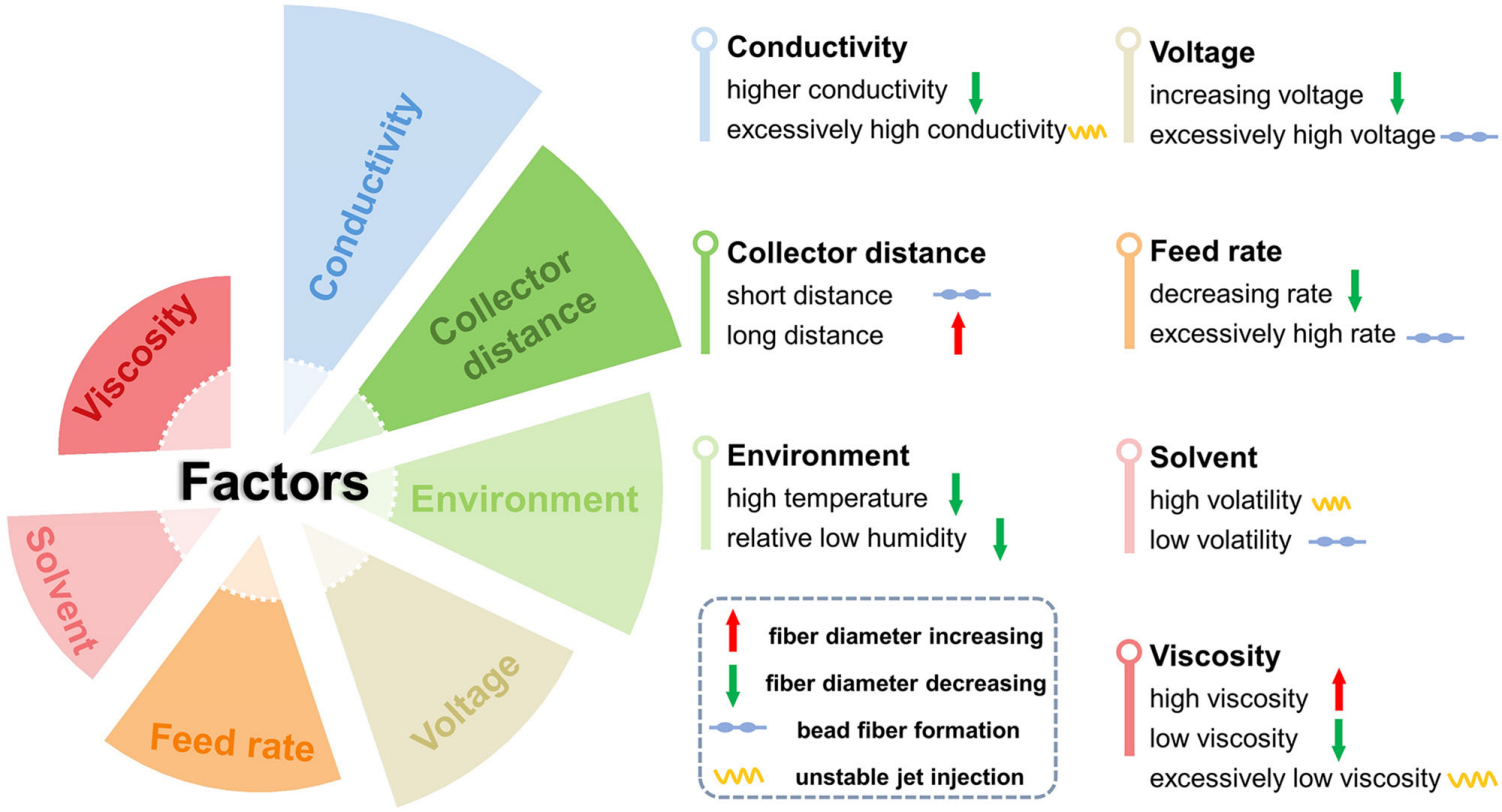
The factors that affect the e‐spin fiber formation and corresponding outcomes

Through precise manipulation of the apparatus and parameters, researchers can fabricate fibrous biomaterials with precisely controlled fiber morphology, diameter, pore size, and spatial organization from microscopic fiber structure to macroscopic fiber arrangement, therefore meeting the requirement of various applications. The main factors that affect the fiber formation including fiber diameter and structure are summarized in Figure 1. In theory, any polymer or melt solution can be directly electrospun into fibers. Organic polymers, such as, biomacromolecules, electroactive polymers, and other functional polymers, are generally suitable electrospinning candidates due to their versatility, functionality, or biodegradability. With the introduction of functional fillers, the electrospun fibers can possess desired mechanical and physicochemical properties for extensive applications.^[^ ^9^ ^]^

**FIGURE 2:**
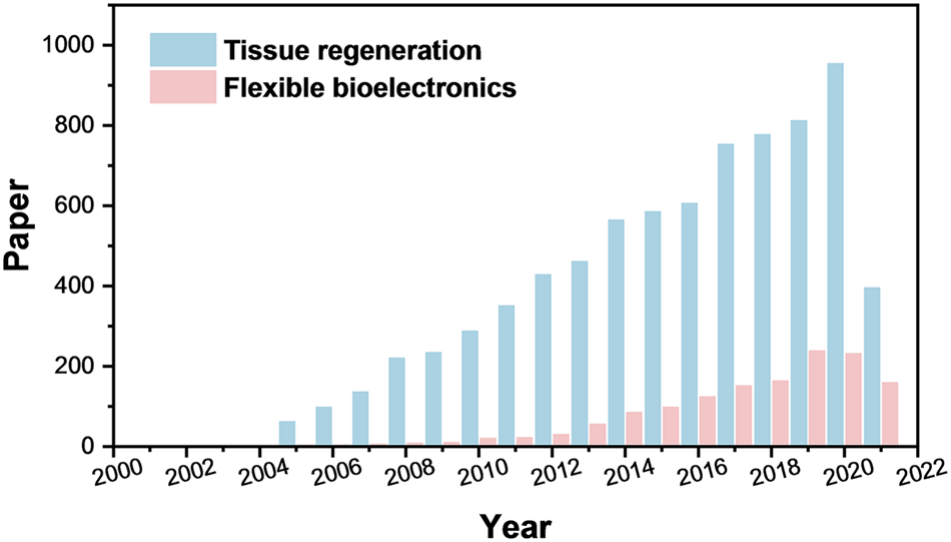
Numbers of academic papers in the last two decades with the keywords “electrospinning/electrospun” and “tissue engineering” or “flexible bioelectronics” from web of science, respectively

In recent decades, application of e‐spin materials in biosensor, bioelectronics, and tissue engineering are prosperously increased (Figure 2). The flourishing development of bioelectronics and tissue engineering requests for more rational design of the structural, mechanical, and physicochemical properties of the fibrous biomaterials. This technology provides a controllable, efficient, and low‐cost alternative for preparing micro/nanofiber with tunable mechanical and electrical features, involving large surface area, high porosity, flexibility, and conductivity. And the fiber assemblies are pivotal in fabricating lightweight and conformable bioelectronics. The applicability of biosafe polymers is ideal to devise supporting scaffolds that directly interact with cells or tissues. In this perspective, we first summarize the state‐of‐the‐art and comprehensive researches on the fabrication of hierarchically structured e‐spin fibers from microscopic fiber structure to macroscopic fiber arrangement. Then, we discuss the latest progress on bioelectronics and tissue engineering applications in which the e‐spin technology has developed by leaps and bounds. Especially, the emerging applications as flexible electronics for bioenergy harvest, biosensing and as tissue regenerative stimulator are highlighted. Finally, we propose the remaining challenges and future perspective to design the materials and devices with desirable functions matching specific applications and also abstract the burning questions on the e‐spin based devices and scaffolds toward real applications.

## 2 STRUCTURAL DESIGN AND FABRICATION OF E‐SPIN FIBERS

**FIGURE 3:**
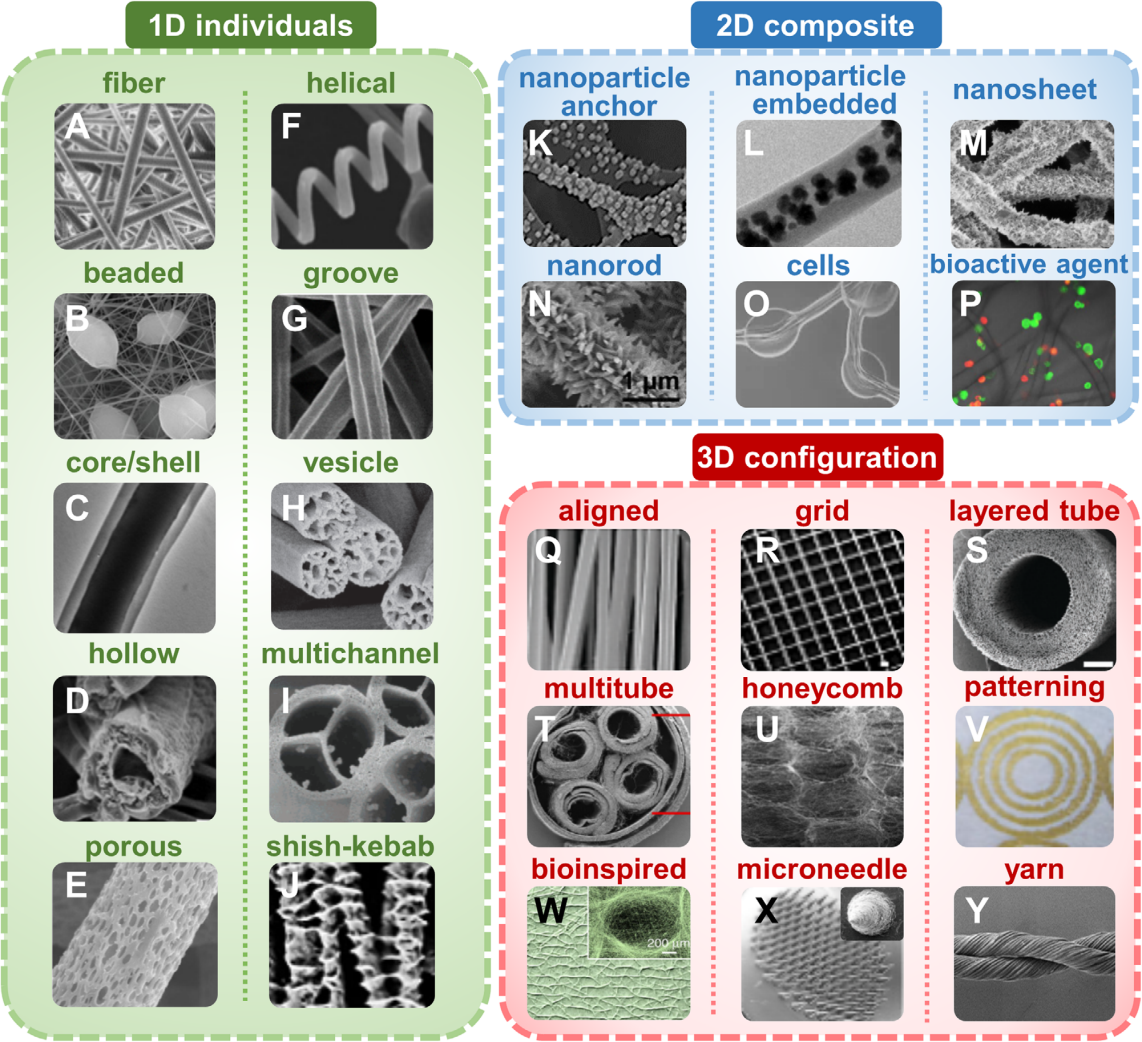
Hierarchically structural design of e‐spin fibers. The diverse structures are divided into three groups: One‐dimension (1D) individual fiber, two‐dimension (2D) composite fiber, and three‐dimension (3D) fiber configuration. Images are some examples of designed fibers which are studied most. (A) solid fiber: Reproduced with permission.^[^ ^27^ ^a]^ Copyright 2020, American Chemical Society. (B) beaded fiber: Reproduced with permission.^[^ ^10^ ^]^ Copyright 2020, Wiley‐VCH. (C) core/shell fiber: Reproduced with permission.^[^ ^6^ ^a]^ Copyright 2018, Elsevier. (D) hollow fiber: Reproduced with permission.^[^ ^11^ ^]^ Copyright 2021, Elsevier. (E) porous fiber: Reproduced with permission.^[^ ^6^ ^a]^ Copyright 2018, Elsevier. (F) helical fiber: Reproduced with permission.^[^ ^11^ ^]^ Copyright 2021, Elsevier. (G) fiber with groove: Reproduced with permission.^[^ ^11^ ^]^ Copyright 2021, Elsevier. (H) fibers with vesicle inside: Reproduced with permission.^[^ ^16^ ^]^ Copyright 2011, Wiley‐VCH. (I) multichannel tubular fiber: Reproduced with permission.^[^ ^12^ ^]^ Copyright 2007, American Chemical Society. (J) fibers with hybrid shish‐kebab structure: Reproduced with permission.^[^ ^15^ ^]^ Copyright 2015, American Chemical Society. (K) nanoparticle anchored fiber: Reproduced with permission.^[^ ^84^ ^]^ Copyright 2020, Elsevier. (L) fibers with embedded nanoparticle: Reproduced with permission.^[^ ^7^ ^]^ Copyright 2020, Elsevier. (M) nanosheet‐grown fiber: Reproduced with permission.^[^ ^3^ ^]^ Copyright 2021, American Chemical Society. (N) nanorod‐grown fiber: Reproduced with permission.^[^ ^19^ ^]^ Copyright 2021, Elsevier. (O) cell‐encapsulated fiber: Reproduced with permission.^[^ ^18^ ^]^ Copyright 2021, American Chemical Society. (P) fiber with bioactive agents (drugs, biomolecules): Reproduced with permission.^[^ ^85^ ^]^ Copyright 2018, American Chemical Society. (Q) aligned fiber: Reproduced with permission.^[^ ^78^ ^]^ Copyright 2019, Wiley‐VCH. (R) grid patterned fiber: Reproduced with permission.^[^ ^67^ ^]^ Copyright 2020, Nature. (S) tubular fiber scaffold: Reproduced with permission.^[^ ^86^ ^]^ Copyright 2020, Wiley‐VCH. (T) multichannel fibrous conduit: Reproduced with permission.^[^ ^27^ ^a]^ Copyright 2020, American Chemical Society. (U) honeycomb‐pattern fiber assembly: Reproduced with permission.^[^ ^49^ ^]^ Copyright 2018, American Chemical Society. (V) complex concentric patterns prepared by localized nanofiber dissolution method: Reproduced with permission.^[^ ^25^ ^]^ Copyright 2014, Wiley‐VCH. (W) macro morphology of biomimetic fiber with a wood‐like structure: Reproduced with permission.^[^ ^2^ ^]^ Copyright 2020, Nature. (X) image of a microneedle array with nanofibrous structure: Reproduced with permission.^[^ ^57^ ^]^ Copyright 2020, American Chemical Society. (Y) electrospun fiber yarn being twisted: Reproduced with permission.^[^ ^87^ ^]^ Copyright 2015, Wiley‐VCH

Most common e‐spin fibers are randomly deposited with smooth and solid structure, which is difficult to satisfy multifarious applications. To fully maximize the versatility of e‐spin, a diversity of intriguing structures has been fabricated by adjusting the composition of polymer solution, designing the architecture of spinnerets and collectors, optimizing e‐spin parameters and environment conditions, and performing post‐treatment if necessary. The hierarchical structures of electrospun fiber show diversity from inner to outer morphology, and from microscopic to macroscopic topography. Therefore, we divide the e‐spin structure into three categories: (1) One‐dimensional individuals (1D): individual nanofibers with/without surface/inner morphology; (2) two‐dimensional composites (2D): fibers with incorporation of functional components to produce a secondary hierarchy; (3) three‐dimensional configuration (3D): the arrangements of fiber alignment or reassembly along three dimensions (Figure 3). Similar with materials fabricated from other techniques, the comprehensive properties of the e‐spin fibers including morphology, viscosity, mechanical, electrical, air permeability/specific surface area performance can be characterized through universal visual microscope, tensile/compression test, thermal/electrical characterization, and porosity quantification, etc.

### 2.1 1D individual fiber

The morphology and structure of one single fiber is the most fundamental structure in the resulting e‐spin fabric. Generally, e‐spin fibers have smooth surface, round cross‐section, and uniform diameter. It is favorable for some applications, but instead hinders the extension of multifunctionality for other ones. In recent studies, e‐spin fiber with diverse and fascinating morphology and secondary structures have sprung up, including beaded,^[^ ^10^ ^]^ core‐shell,^[^ ^6^ ^a]^ hollow,^[^ ^11^ ^]^ multi‐channel,^[^ ^12^ ^]^ helical,^[^ ^11^ ^]^ porous,^[^ ^6^ ^a]^ and internal cavity.^[^ ^13^ ^]^ Generally, these modified structures can be fabricated by adjusting the e‐spin parameters or changing numbers and configurations of the spinnerets.

Through adjusting the viscosity of polymer solution, the structure can be easily converted between beads, beaded fibers, and solid fibers. The formation of beaded fiber is mainly attributed to that the low viscosity causes instable fluid jet when exposed to a high voltage. This kind of structure is once considered as a dissatisfying structural defect during e‐spin. More recently, it is found that the beaded fibers with swelling‐like structure have increased surface roughness, which can reinforce the interfacial properties to bring unconventional biological effects such as induction of cell differentiation.^[^ ^14^ ^]^

Fibers with complex internal or external structures have distinctive anisotropy, high surface area, and transport efficiency. Among diverse modified e‐spin technologies, coaxial electrospinning is a straightforward method to synthesize fibers with various nano/microstructures. Utilizing more than one spinneret loaded with different solutions in concentric configuration, coaxial, and immiscible fibers can be obtained. Core‐shell, hollow and multichannel structural fibers are generated with the increment of structural diversity of the spinnerets. Via changing the composition and proportion of e‐spin solution in the core/shell spinnerets, fibers with different components and functions can be fabricated, which combine advantages and characteristics of each component.

Porosity can drastically increase the specific surface area and lighten the weight of the resultant fibers. Cooling‐, vapor‐, and liquid‐induced phase separation between polymer and solvent can successfully produce porous fibers.^[^ ^1^ ^]^ Using two kinds of polymers with distinct properties such as viscoelasticity or conductivity, fibers with helical alignment can be fabricated. For example, Zeng et al. fabricated polymeric helical/hollow nanofiber by tri‐fluid electrospinning.^[^ ^11^ ^]^ The rigid thermoplastic polymer (cellulose acetate) and flexible thermoplastic polymer (polyurethane) were selected to generate helical sheath due to their different bending ability. And polyvinylpyrrolidone was adopted as the scarified core fluid for subsequent hollow‐structure formation.

Other fibers with unique structure have also been reported. For instance, shish‐kebab fibers with hierarchically ordered feature was realized by guiding the oriented crystal growth.^[^ ^15^ ^]^ Besides, vesicles inside the fibers were obtained via oil‐in‐water emulsion e‐spin strategy.^[^ ^16^ ^]^

### 2.2 2D composite fibers

Distinct from 1D individuals, 2D composite fibers with secondary structures are usually fabricated by directly blending functional moieties including nanoparticles, drugs, bioactive molecules, cells, and virus into polymers. Besides the generated changes in fiber morphology similar with 1D individual, the composite fibers emphasize the additional functions after the moieties’ incorporation. These filler‐embedded fibers often exhibit intriguing properties and show advances in specific applications. For example, multimetallic nanoparticles with up to 8 dissimilar elements PtPdCoNiFeCuAuSn in single‐phase nanoparticles were directed incorporated into carbon nanofibers, realizing ≈100% ammonia conversion.^[^ ^17^ ^]^ Inspired by the structure of lymph vessels, Nie et al. fabricated a cell‐laden fiber in the shape of beads‐on‐a‐string using coaxial e‐spin.^[^ ^18^ ^]^ The cells encapsulated in the fiber preserved high viability and capability to secrete immune molecules just like the real lymph vessel. It was beneficial for the assay of human immune response in vitro and functional replacement in vivo. In situ growth and post‐treatment to generate other components on the fiber is another general strategy to fabricate 2D composite fibers with hierarchical morphology and versatile functions. Liu et al. fabricated FeOOH nanoneedles doped PVDF nanofibers by immersing PVDF nanofibers in the ferric chloride/hydrochloric acid solution, followed by a hydrothermal reaction.^[^ ^19^ ^]^ Using a similar method, Zhu et al. fabricated polyacrylonitrile@CuS fibrous films with photothermal CuS nanosheets aligned on the fibers after an in situ sulfurization. It remarkably increased the specific surface area for over 4 times and displayed a high‐rate evaporation under solar irradiation.^[^ ^3^ ^]^

### 2.3 3D fiber configuration

The arrangement of e‐spin fibers fabricated from 1D individuals and/or 2D composite fibers also exhibits marvelous diversity. As mentioned above, the morphology of individual fiber depends on the spinneret setting, whereas the structural diversity of fiber is greatly influenced by the collector type, which determines the alignment and distribution of e‐spin fibers. In general, the fibers are randomly oriented when a traditional conductive grounded plate is applied, mainly due to the bending instability of the emanated jet during the e‐spin process.^[^ ^20^ ^]^

However, in specific applications, fiber with well‐aligned structure is beneficial for directing cell behavior, simulating biotissue, optimizing mechanical properties and improving electrical conductivity. By engineering the collector apparatus including the addition of mechanical, electrical, and magnetic force or employment of near‐field electrospinning, ordered fibers can be harvested. Elizabeth et al. systematically summarized existing methods to induce e‐spin fiber alignment, involving rotating collector setup, gap electrospinning setup between a pair of parallel metallic plates, using a metallic ring with a conductive pin in the center, magnetic field‐assisted electrospinning setup, auxiliary electrode setup, and centrifugal electrospinning.^[^ ^21^ ^]^ Moreover, the enhancement of conductivity of e‐spin fluid via adding salt into polymers can effectively improve the consistency of fiber orientation.^[^ ^22^ ^]^

In addition to aligned fibrous structures, biomaterials with patterned micro/nanostructures show unique interaction with and influence on cells/tissues. Recently, e‐spin has been modified to produce patterned films, such as direct utilization of patterned templates as collector, self‐assembly of electrospun fibers on the collector, and post‐treatment of the electrospun films. Some reviews have summarized regularly patterned configurations for fabrication of patterned fibrous films, including grid scaffold, round, woven, rectangular, protruding, or reentrant structures.^[^ ^8^ ^]^ In a typical study, a unique honeycomb‐patterned poly(vinyl alcohol) (PVA) nanofibrous architecture was obtained through the self‐assembly of wet fibers driven by the interaction of electrostatic repulsion and surface tension.^[^ ^23^ ^]^ Kim et al. designed a piezoelectric fibrous substrate with repetitive pattern of gradient alignment to investigate the cell response toward topological and electrical cues.^[^ ^24^ ^]^ This pattern was deposited on the manual collector that was prepared by alternately connecting conductive wire and nonconductive tape. Progressively, post‐treatments such as local dissolution,^[^ ^25^ ^]^ weaving and twisting of the as‐spun fiber into yarns represent other alternate ways to fabricate fibers with versatile patterns.^[^ ^26^ ^]^

3D fibrous constructs are promising in practical applications such as tissue engineering owing to the closer reproduction of natural tissue structure. Currently, despite of great difficulties, some progress has been made in constructing 3D e‐spin macrostructures. Among various strategies, the most straightforward approach is to deposit the fibers along the third dimension directly. By engineering the collectors, the stacked fibers can gradually form a 3D construct with thickness of several‐centimeters.^[^ ^22^ ^]^ Post‐treatment of the as‐spun 2D fibers can also realize 3D construction, for example, layer‐by‐layer e‐spin, weaving, folding, freeze drying of 2D mat fragments. Typically, by using thermo‐responsive polymers or shape‐memory polymers, the resultant fibrous films can deform and self‐bend to form 3D tubular structure once the temperature was changed.^[^ ^27^ ^]^ Gas‐foaming is another strategy to generate 3D fibrous foam,^[^ ^28^ ^]^ which realizes physical expansion of the fibrous films through gas generation from chemical reactions. The gas‐foaming process can facilitate the encapsulation of therapeutic drugs or other active moieties for further applications.^[^ ^28^ ^a]^

## 3 APPLICATIONS OF E‐SPIN FIBER IN FLEXIBLE BIOELECTRONICS AND TISSUE ENGINEERING

E‐spin fibers have been applied in various fields. Of all the proposed applications, tissue engineering has become one of the most valued one in recent years. And flexible bioelectronics has been considered as a cutting‐edge and rapid developing research area for intelligent healthcare, in which e‐spin can be used to fabricate electronic modules and substrates. The performance and applications of various e‐spin fiber‐based materials emerging in flexible bioelectronics and tissue engineering are listed in Table 1.

**TABLE 1:** Electrospun fiber‐based materials for flexible bioelectronics and tissue engineering

**Entry**	**Composition**	**Fiber structure**	**Properties and functions**	**Applications**	**Ref**.
Flexible bioelectronics	Core: PVDF‐BTO Shell: PVDF‐GO	Core‐shell	Piezoelectricity, conductivity, sensitivity of 10.89 ± 0.5 mV kPa^–1^	Human motion monitoring and tactile imaging	[7]
	PVA/Polyurethane/Au	Solid, random	Ultrathin nanomesh without sensory interference	Finger force monitoring	[29]
	PU	Solid, random	Flexibility, no interference	Pulsing cardiomyocytes monitoring	[30]
	Outer shell: PVA Inner shell: DA Core: PVDF	Core‐shell, aligned	Humid sensitivity and selectivity	Mental sweating monitoring	[31]
	PVDF/BTO	NPs‐embedded, random	Lightweight, sensitivity of 3.95 V N^−1^	Physiological monitoring	[32]
	Core: PVDF Shell: hydroxylamine hydrochloride	Core/shell, random	Self‐orientated nanocrystals, enhance β‐phase of PVDF	Detection of cardiovascular micropressure	[33]
	Core: PVDF Shell: DA	Core/shell, random	Enhance β‐phase of PVDF, soft, piezoelectricity	Detection of diaphragm motions and blood pulsation	[34]
	Silver‐doped PVDF	Aligned	Flexibility, enhanced piezoelectricity than random one	Respiratory monitoring	[35]
	PVDF‐TrFE, PU, PVDF‐HFP	Random	Triboelectric, piezoresistive, thermoresistive sensing	Human motion and breathing sensors	[36]
	P(VDF‐TrFE)/BTO	NPs‐anchored, random	Self‐powered, 84 V, 1.32 μA	Implantable vagal neuromodulation stimulator	[38]
	PVDF‐TrFE	Aligned	Piezoelectricity, electromechanical stimulation, ion channel modulation	Piezo‐bioelectronics	[39]
	PCL/gelatin	Random	Biomimicking of heart matrix, porous, penetrative	Cell electrical activity recording and therapeutic control	[40]
	BTO crystals	Solid, random	Flexibility, fast response time of 80 ms	Piezoelectric sensors	[76]
	Silica	fiber fragment	High robustness, transparent, conductivity of 3.93 S m^–1^	Pulse and handwriting detecting	[77a]
	Carbon nanotube (CNTs)	Yarn	Flexibility, 3D‐printed, temperature sensitivity of 1.95%°C^−1^	Wearable temperature sensor	[83a]
Tissue engineering	Inner layer: HAp‐loaded gelatin Outer layer: antibacterial agent‐loaded PCL	Random inner and aligned outer layer	Enhanced osteogenic and antibacterial effects, macrophages polarization	Bone regeneration	[41]
	MSN‐based PCL/gelatin	Particle‐embedded, random	Dual‐delivery for increased bone formation and inhibited bone resorption	Bone regeneration	[47a]
	PCL/HAp	Honeycomb‐like	Differentiated bone cells without chemical factor	Maxillofacial repair in bone regeneration	[49]
	MgO‐loaded PLA/gelatin	NPs‐embedded. random	Biodegradable, elevated mechanical, antibacterial, and osteogenic properties	Periodontal tissue regeneration	[64]
	Gelatin/PLGA	3D‐printing scaffolds, latticed	Chondrocytes‐laden, good elasticity, and water‐induced shape memory	Cartilage regeneration	[83b]
	PCL/poly(3‐hydroxybutyrate) (PHB)/PANi	Bioactive molecular‐laden	Enhanced piezoelectricity, prolonged drug release, enhanced osteogenesis, and mineralization	Bone tissue engineering	[85]
	PVDF/FeOOH	Nanorod on fiber	Ultrasonic‐driven piezoelectricity and ion release, neural differentiation	Neural tissue engineering	[19]
	SMPs	Aligned, 4‐channel tubular conduit	Bioinspired, degradable, cell‐laden	Peripheral nerve regeneration	[27a]
	Gelatin methacrylate (GelMA)	Aligned conduit	Inducing neural differentiation, inhibiting the glial scar formation	Spinal cord regeneration	[78]
	PCL	Aligned	Functionalized with gradient concentration of NGF, similar performance with autograft	Sciatic nerve regeneration	[86]
	PCL/silk fibroin/CNTs	Interwoven aligned	Promoted cell maturation and endothelialization	Artificial 3D cardiac anisotropy for cardiac tissue regeneration	[26a]
	PLGA, PVDF, cellulose	Aligned and helix yarn	Highly stretchable, promoted myogenic differentiation	Various tissue engineering	[26b]
	CNTs sheets	Superaligned	Efficient electrotonic propagation, reduced signal dispersion	Myocardial resynchronization in cardiac tissues	[54]
	GelMA	Random	Tissue‐adhesive patch, optimized mechanical and conductive properties, restore electromechanical coupling	Cardiac tissue regeneration	[77b]
	PU	Aligned array onto a latticed gauze fiber	Self‐pumping the biofluid, faster re‐epithelialization, and collagen formation	Wound healing for skin regeneration	[44]
	PCL/F‐127	3D scaffold with radially or vertically aligned nanofibers	Enhanced re‐epithelialization or granulation tissue formation in the diabetic wound	Diabetic wound healing	[58]
	PLGA/fish collagen	Random, aligned and latticed	Better healing effect and immunomodulatory properties for the aligned one	Wound healing for skin regeneration	[81]
	PLGA/PCL	Rolling up into tubular scaffolds	Three cell lineages‐laden to form a biomimetic vessel, controllable shape during biodegradation	Vascular tissue engineering	[45]
	PCL	Double layered tube Inner: random Outer: orientated	3 mm diameter, endothelial progenitor cells and differentiation of MSCs into smooth muscle cells	Vascular tissue engineering	[72]
	PCL/GelMA	Self‐rolled from 2D surface into 3D tubular shape at 37°C	Desirable endothelial cell attachment, deformation properties	3D endothelialization	[79b]

### 3.1 Flexible bioelectronics

Flexible bioelectronics including wearable and implantable devices can be used for intelligent and personalized healthcare through sensing, monitoring and therapy. For recent years, flexible bioelectronics have been developed for the detection and recording of biophysical signals (e.g., stress, temperature, optics, and movement), biochemical signal (e.g., gas, biomolecules, and metabolites), and electrophysiological signals as daily health surveillance. Biocompatible and structurally matchable bioelectronics can deliver electric stimulation for therapeutic purpose especially at the neural network interface. As the ultimate goal, it is expected that a bioelectronic device can integrate the function of recording and self‐feedback therapy, such as on‐demand electric stimulation or programmed delivery of therapeutic molecules. Compared with traditional rigid films and devices, the flexible bioelectronic systems matching natural tissues offer promising advantages for real‐time monitoring over a long period without influencing normal body action and tissue function.

**FIGURE 4:**
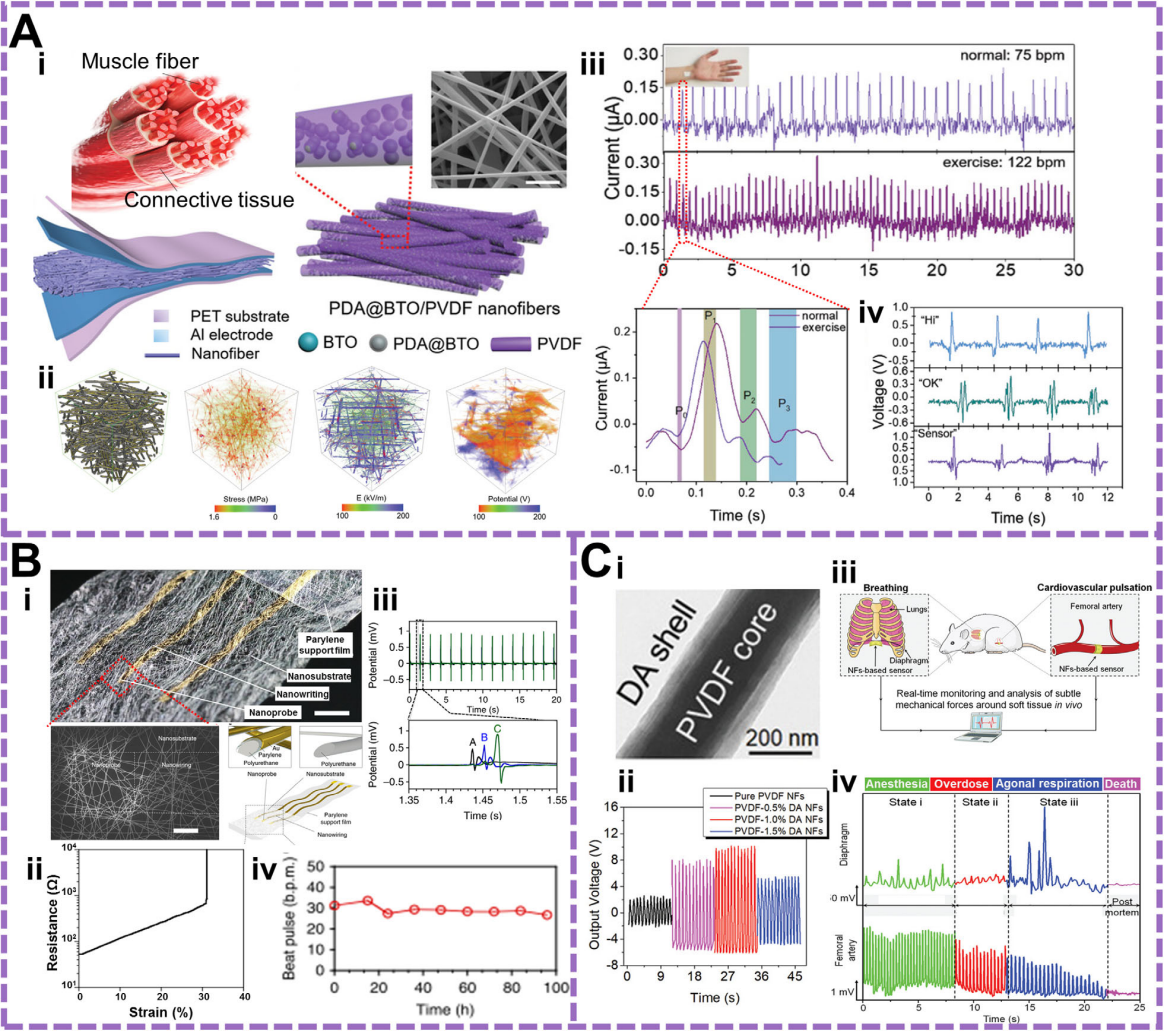
Applications of the e‐spin based materials in flexible bioelectronics. (A) Muscle fibers inspired piezoelectric wearable sensor for physiological monitoring including pulse recording and voice recognition. (i) Structure design and morphology of the piezoelectric fibers composed of PVDF and polydopamine (PDA) functionalized BTNPs. (ii) Phase‐field simulations of piezoelectric fibers. From left to right is the structural modeling, stress distribution, electric field distribution and piezoelectric potential distribution. (iii) Real‐time monitoring of pulse for static status and dynamic status after exercise. The red line circle is the enlarged profile in one pulse. (iv) Dynamic output profile for voice recognition when saying different words (Hi, OK, and Sensor). Reproduced with permission.^[^ ^32^ ^]^ Copyright 2021, Wiley‐VCH. (B) Ultrasoft nanomesh sensors monitoring dynamically pulsed cardiomyocytes. (i) Optical image and corresponding image of nanomesh device. (ii) Resistance change of nanomesh under tensile strain. (iii,iv) Electrophysiological monitoring of cardiomyocytes using the nanomesh sensors. Reproduced with permission.^[^ ^30^ ^]^ Copyright 2019, Nature. (C) Core/shell piezoelectric nanofiber as biomedical sensors for monitoring cardiovascular walls. (i) TEM image of the individual core‐shell fiber. (ii) Piezoelectric output from the nanofibers with different dopamine concentrations. (iii,iv) Schematic of implantation of the nanofiber‐based sensor and corresponding voltage signals induced by diaphragm motions and blood pulsing when the mouse was at different physiological states. Reproduced with permission.^[^ ^34^ ^]^ Copyright 2020, Wiley‐VCH

To this end, e‐spin technology possessing low‐cost and large‐scale priority stands out against other fabrication strategies to provide the flexible, stretchable, light‐weighted, conductive, degradable, and biocompatible functional constituents in the field of flexible bioelectronics. Flexible and stretchable fiber especially 2D film can be facilely fabricated, acting as the conformal substrate or electrode. It can seamlessly contact with biological tissues, such as, skin to satisfy the demands of comfort and reliable sensing. The ultra‐flexibility of electrospun fiber can also enhance the sensitivity of devices to perceive weaker physiological signals such as, the subtle vibration of vocal cords and the beating of pulses (Figure 4A).

Someya et al. developed a multilayered nanomesh pressure sensor composed of ultrathin polyurethane e‐spin passivation layer, a parylene‐coated polyurethane intermediate layer and Au nanomesh electrode layers prepared using electrospun PVA nanofibers as the sacrificial layer.^[^ ^29^ ^]^ For monitoring finger manipulation, the nanomesh sensor with a whole thickness of 2 mm realized an ultrahigh sensitivity of 0.141 kPa^–1^ under 1 kPa and 0.01 kPa^–1^ above 10 kPa. When attached on the fingertip, the sensors would not affect the natural feeling of the skin, which improved the accuracy and comfort of sensory process. Furthermore, the same group reported similar ultrasoft nanomesh with proper mechanical and electrical properties to monitor the dynamic pulse and field potential of in vitro cultured cardiomyocytes (Figure 4B).^[^ ^30^ ^]^ This device comprised three layers of e‐spin polyurethane fiber, parylene fiber, and sacrificial e‐spin template derived Au electrode and can be stretched to 30% strain while preserving high conductivity. The reliable and intimate contacts between the e‐spin fiber derived electrode and the cardiomyocytes ensured its sensitive measurement, and the porosity of the e‐spin structure allowed efficient supply of nutrients thus facilitating continuously measurement without damage to the cells.

By wrapping PVA onto piezoelectric PVDF/dopamine (DA) to form shell/core nanofiber, Li et al. fabricated a humidity‐actuated flexible nanogenerator with high sensitivity.^[^ ^31^ ^]^ This device went through mechanical deformation under weak humidity fluctuations to produce electric power through piezoelectric fibers. Importantly, the authors proposed that this device could detect subtle humidity fluctuation induced by various mental sweating, thus realizing inverse tracking of the mental state change. This work paves the way for the development of wearable flexible device for healthcare monitoring and mental state analysis. Up to now, functional e‐spin fiber‐based devices have been exploited to monitor physiological signals in a real‐time such as, voice recognition,^[^ ^32^ ^]^ blood pressure,^[^ ^33^ ^]^ pulse wave,^[^ ^34^ ^]^ and respiration.^[^ ^35^ ^]^ All these commendable outcomes verify the e‐spin as a conceivable strategy for realizing precision medicine and telemedicine in the future.

Electrospun fibers can also be utilized for preparation of implantable bioelectronic devices. Through performance optimization such as surface modification at the interface, the dynamic tissue activity can be accurately monitored. Feng's group developed a sensor from PVDF/dopamine (DA) core/shell piezoelectric fiber and implanted it on the diaphragm membrane for detecting cardiovascular diseases.^[^ ^34^ ^]^ The piezoelectric effect of PVDF was significantly enhanced by the dipolar interactions between PVDF and DA during e‐pin process, thus improving the sensitivity toward slight motion of angiocarpy (Figure 4C). In the newest researches, flexible bioelectronics based on piezoelectric/triboelectric effects can convert the biomechanical energy such as heartbeat, vessel expansion, joint slipping, and into electricity, which realized wireless and self‐powered sensing for personalized healthcare.^[^ ^36^ ^]^ With the self‐powering property, e‐spin piezoelectric fiber is regarded as an ideal candidate for implantable flexible electric stimulator. Successful attempts have been made in skin,^[^ ^37^ ^]^ vagus nerve,^[^ ^38^ ^]^ tendon,^[^ ^39^ ^]^ and heart tissue.^[^ ^40^ ^]^ It is expected to substitute part of traditional pharmaceuticals toward clinical significance. Nevertheless, the most existing e‐spin flexible bioelectronics are not biodegradable, thereby requiring a second operation to be taken out when implantation is needed. Moreover, in the further development, the problem of long‐life durability of the fiber‐based devices caused by the instable electrospinning preparation also urgently needs to be overcome.

### 3.2 Tissue engineering

Tissue engineering and regenerative medicine aim to provide tissue substitutes, which involves main elements of cells, scaffolds, and inductive factors to accelerate tissue repairing. It is critical to construct a proper biophysical and biochemical microenvironment for bridging natural tissues and activating cells. Compared with other typical materials such as, coating membranes and hydrogels, nanofibrous materials can expediently imitate the natural extracellular matrix (ECM) of different tissues in terms of structure, biochemical composition, mechanical property, and electrical activity, which can not only serve as supportive substrate but also regulate cell behaviors with the advantages of efficiency, simplicity, and low‐cost setup.

To meet the demands of a target tissue, the architectural, physiochemical, and mechanical properties of the corresponding native ECM should be fully considered for the fabrication of biomimetic scaffold with e‐spin. For example, bone has spongy architecture and outer aligned collagen fibril, peripheral nerve possesses myelin sheath with multichannel, heart exhibits aligned cardiomyocytes and interwoven myocardium of wall, and vessel displays multilayered structure composed of different cell lines. These complicated anisotropies are difficult to be duplicated by conventional fabrication methods. The scaffolds with aligned, core/shell, porous features from 2D to 3D assembled e‐spin fibers have been fabricated to match the tissue organization and cellular adaptation. Up to now, extensive applications of e‐spin fibrous scaffolds have been investigated for the regeneration of functional tissues including hard tissues of bone,^[^ ^41^ ^]^ and cartilage,^[^ ^42^ ^]^ and soft tissues such as nerve,^[^ ^27^ ^a]^ heart,^[^ ^43^ ^]^ skin,^[^ ^44^ ^]^ blood vessel,^[^ ^45^ ^]^ skeletal muscle.^[^ ^46^ ^]^

#### 3.2.1 Hard tissue engineering

As a typical hard tissue, bone can be considered as a complex hierarchical architecture comprising inorganic mineralized carbonated hydroxyapatite (HAp) and organic type‐I collagen fibrils with resident osteoblasts and osteoclasts, which modulate the dynamic remodeling of bone for maintaining mechanical strength and calcium homeostasis. Cartilage is a flexible connective tissue consisting of type‐II collagen, proteoglycan, and cartilage cells. The modulus of elasticity of bone and cartilage is in the range of 1–2 GPa and 5.7–6.2 MPa, respectively.

Considering the mechanical toughness and high mineral proportion of natural bone tissue, e‐spin scaffolds for bone regeneration are commonly fabricated by reinforcing the biodegradable polymers (e.g., polycaprolactone (PCL), poly(lactide‐coglycolide) (PLGA), polylactic acid, and poly(L‐lactic acid) (PLLA), and poly(l‐lysine)) with inorganic phase (e.g., HAp, silicate and BTNPs).^[^ ^41^, ^47^ ^]^ An ideal 3D electrospun scaffolds should resemble the microscopic properties of ECM including the mechanical, structural, and component properties.^[^ ^48^ ^]^ The high mechanical toughness and hierarchical architecture can withstand the changing mechanical load during bone healing, and is also instructive to promote cell osteogenesis.^[^ ^42^ ^]^ Via integrating electrospraying technique, Alejandro et al. elaborated an e‐spin 3D scaffold composed of alternating layers of PCL and HAp with a honeycomb‐like morphology, which directed the osteoblast transition from embryonic cells without differentiation additives.^[^ ^49^ ^]^

Additionally, bioactive molecules are integrated into e‐spin fibers to further reinforce the regeneration and cell induction capacity.^[^ ^18^, ^41^ ^]^ To this end, a composite scaffold could deliver multifunctional agents to balance bone formation and resorption, thus shortening the recovery time.^[^ ^47^ ^a]^ The integration of growth factors is another strategy to improve e‐spin performance including bone morphogenetic proteins, vascular endothelial growth factor, fibroblast growth factor, and transforming growth factor beta.^[^ ^42^ ^]^

**FIGURE 5:**
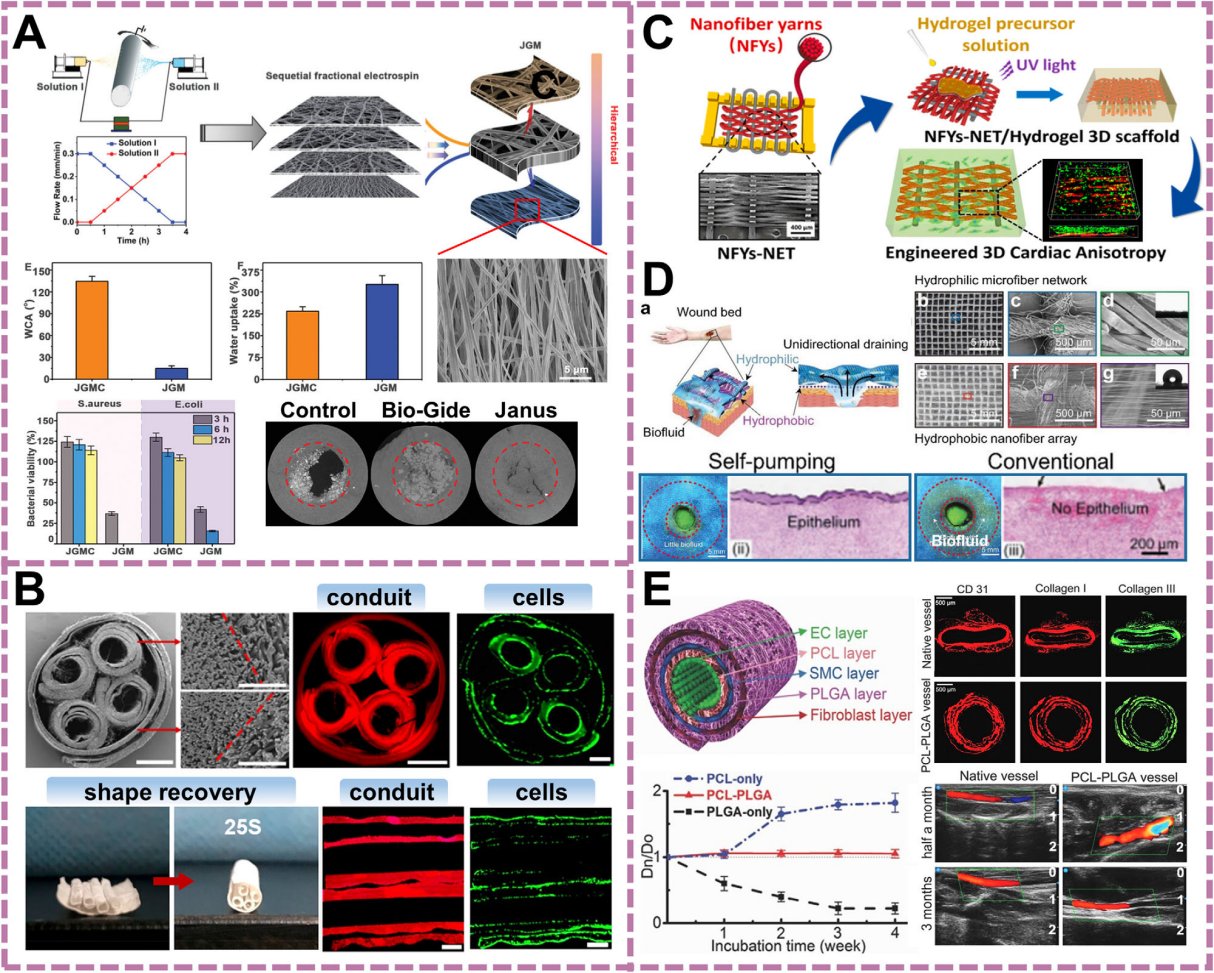
Applications of the e‐spin based materials in tissue engineering. (A) A Janus‐type e‐spin guided film with the functions of advanced bone regeneration osteoimmunomodulatory capability. Reproduced with permission.^[^ ^41^ ^]^ Copyright 2020, Wiley‐VCH. (B) Interwoven aligned PCL/silk fibroin/CNTs fiber yarn/hydrogel hybrid scaffolds for engineering 3D cardiac anisotropy. The microstructure was shown to modulate cellular alignment and enhance cardiomyocytes maturation, which suggested potential in cardiac tissue engineering. Reproduced with permission.^[^ ^26^ ^a]^ Copyright 2017, American Chemical Society. (C) Bioinspired four‐channel NGC with uniaxial guidance based on shape memory fiber for repairing sciatic nerve defects. Reproduced with permission.^[^ ^27^ ^a]^ Copyright 2020, American Chemical Society. (D) A self‐pumping dressing composed of hydrophilic microfiber network and hydrophobic nanofiber array for accelerating wound healing via removing excessive biofluid around wound. Reproduced with permission.^[^ ^44^ ^]^ Copyright 2019, Wiley‐VCH. (E) Artificial fibrous vascular scaffold with self‐adjusting capability and triple‐layered tubular structure made of PCL and PLGA. The distinct degradation rate of PCL and PLGA helped to keep the shapes of blood vessel, which realized a balance between degradation of the implanted vascular substitute and new tissue regeneration. Reproduced with permission.^[^ ^45^ ^]^ Copyright 2017, Wiley‐VCH

More recently, the anti‐inflammatory and immunomodulatory ability of the e‐skin fibrous scaffold is found. Wang et al. proposed a Janus‐structured nanofibrous membrane for skull bone regeneration (Figure 5A).^[^ ^41^ ^]^ The inner e‐spin layer made of HAp and gelatin promoted osteogenesis and the proliferation of osteoblasts, while the aligned PCL fiber loaded with antibacterial components served as the outer layer to suppress bacterial infection, suggesting a favorable osteoimmune environment.

#### 3.2.2 Soft tissue engineering

Soft tissues, including muscle, nerve, skin, vascular tissue, and fascia at the interface of tissues, generally functions as a locomotive apparatus and have considerable elasticity. Hence, e‐spin fiber with controllable mechanical strength is easy to match the architecture and mechanical property of the soft tissues for promoting the tissue repairing.

As for nerve and muscle tissues (including cardiac, skeletal, and smooth muscle), the oriented alignment of ECM plays an indispensable role in guiding cell alignment and constructing the rapid cell connection for signal transmission. E‐spin scaffolds with aligned structure can match the requirement of the architecture.^[^ ^50^ ^]^ Many studies have demonstrated that the unidirectionally oriented fibrous scaffolds had tendency to enhance cell adhesion, proliferation, alignment and maturation of muscle cells (including cardiomyocyte and myoblast), as well as the neurite polarization and extension (Figure 5B).^[^ ^26^ ^a,^ ^51^ ^]^ Along with the topographic cues, other physical cues for cell modulation can also be incorporated into fibers.^[^ ^52^ ^]^ For instance, electroactive components such as conductive graphene oxide,^[^ ^53^ ^]^ carbon nanotube,^[^ ^54^ ^]^ polyaniline (PANi),^[^ ^46^ ^]^ and piezoelectric PVDF,^[^ ^24^ ^]^ were integrated into fibers, resulting in a more efficient electric stimulation toward electroactive cells.^[^ ^55^ ^]^ For in vivo application especially for repairing myocardial infraction and peripheral nerve gap damage, the interventional scaffolds are commonly assembled into 3D architecture to achieve better curative effect.^[^ ^27^ ^a,^ ^56^ ^]^ By using shape memory polymers, the transition from 2D to 3D structure can be conveniently realized without complicated post‐treatments. For instance, Wang et al. reported a four‐channel nerve guidance conduit using thermal‐sensitive poly(lactide‐*co*‐trimethylenecarbonate) e‐spin fibers (Figure 5C).^[^ ^27^ ^a]^ The four channels and the outer conduit were formed from fibrous planar structure to tubular architecture via self‐shaping process initiated by physiological temperature at around 37°C, which ultimately promote angiogenesis and achieved long‐gap peripheral nerve regeneration (10 mm).

With the unique features, bioengineered substitutes based on e‐spin fibers are also regarded as promising therapeutic alternatives for curing skin and vascular in chronic or acute wounds/burns and cardiovascular‐related disease. The high specific surface area and porosity of e‐spin guarantee the gaseous exchange, nutrient transport, functional molecule delivery, and absorption of excessive exudates, which interactively enhance cell migration and infiltration. In order to facing complicated clinical practice, the issues of anti‐inflection/inflammation, alleviation of scar production, wound healing of cutaneous defects of diabetics and skin cancer patients should be comprehensively taken into consideration. Wang's group demonstrated a Janus nanofibrous dressing with opposite hydrophilicity for fastening wound healing.^[^ ^44^ ^]^ The outer hydrophobic nanofiber and inner hydrophilic microfiber produced a draining force to extract biofluid, thus protecting the healing wound from excessive wettability and fastening the wound closure and re‐epithelialization (Figure 5D). When integrated with antimicrobial peptide, the Janus fiber dressing can also eradicate biofilms in a type 2 diabetic infection model to restore ulcer area.^[^ ^57^ ^]^ 3D porous scaffolds with aligned fibers could promote the infiltration and proliferation of repairable cells (e.g., fibroblasts and keratinocytes) which is promising for deep wound recovery within a short period.^[^ ^58^ ^]^ By incorporating CD40 antibody into PLLA electrospun fibers, Cui et al. fabricated a multifunctional scaffold that stimulated the immune response to eliminate cancer and allowed MC3T3‐E1 cells proliferation to promote trauma recovery.^[^ ^59^ ^]^

Vascular scaffold is another extensively studied hotpot in soft tissue engineering. Native blood vessel is in a tubular shape which consists of adventitia populated by fibroblasts, media populated by smooth muscle cells and intima populated by endothelial cells.^[^ ^60^ ^]^ Hence, the tubular morphology with multilayered wall has attracted most attention because of the well‐mimicking of natural blood vessels. Besides structure, appropriate porosity is another essential element for artificial vessel to support blood flow without leakage and smoothen the cell infiltration.^[^ ^61^ ^]^ Although the engineered vascular scaffolds have been continuously optimized in function, thrombosis, and intimal hyperplasia happens inevitably due to the mismatched mechanical properties between collapsed scaffolds and nascent tissues, which seriously hinders its clinical transformation.^[^ ^62^ ^]^ To solve these problems, Zhao et al. reported a small‐diameter vascular graft (<6 mm) by combing e‐spin PCL and nonantigenic decellularized rat aorta.^[^ ^63^ ^]^ To avoid the negative consequence, rapamycin as a delivered drug was blended‐electrospun with PCL to effectively reduced intimal hyperplasia. Another challenge that e‐spin vascular scaffolds encounter is the difficulty in structure maintenance. Collapsed vascular scaffold will block blood flowing and considerably hinder the new vessel regeneration. To avoid this happens, Jiang et al. selected PCL and PLGA as the inner and outer layer of a multilayered artificial vessel.^[^ ^45^ ^]^ The distinct degradation rate of the two polymers showed excellent tubular shape maintenance and high patency as the native vessels after 3‐months implantation (Figure 5E). These feasible strategies provide referable guidelines for ideal electrospun vascular substitute with shape adaptivity, matched mechanical dynamics, and hemocompatibility.

Additionally, e‐spin scaffolds have also been investigated for the potential use in the repair of periodontium,^[^ ^64^ ^]^ tendon,^[^ ^39^ ^]^ muscle,^[^ ^65^ ^]^ hair,^[^ ^66^ ^]^ corneal stroma,^[^ ^67^ ^]^ oesophagus,^[^ ^68^ ^]^ and bladder.^[^ ^69^ ^]^ These results highlight the universality of electrospun materials for the reconstruction of various tissue impairment. Despite of the gratifying progress, most studies still only concentrate on the fabrication of different e‐spin scaffolds using various polymers, which is preliminary for the practical translation toward the clinics. Due to the difficulty in reduplicating the e‐spin process, obstacles may be brought forth about the in‐depth investigation on biological mechanism when compared with other kind of materials, and large‐scale production is hardly to be realized for high‐throughput screening of the materials.

## 4 PERSPECTIVES

**FIGURE 6:**
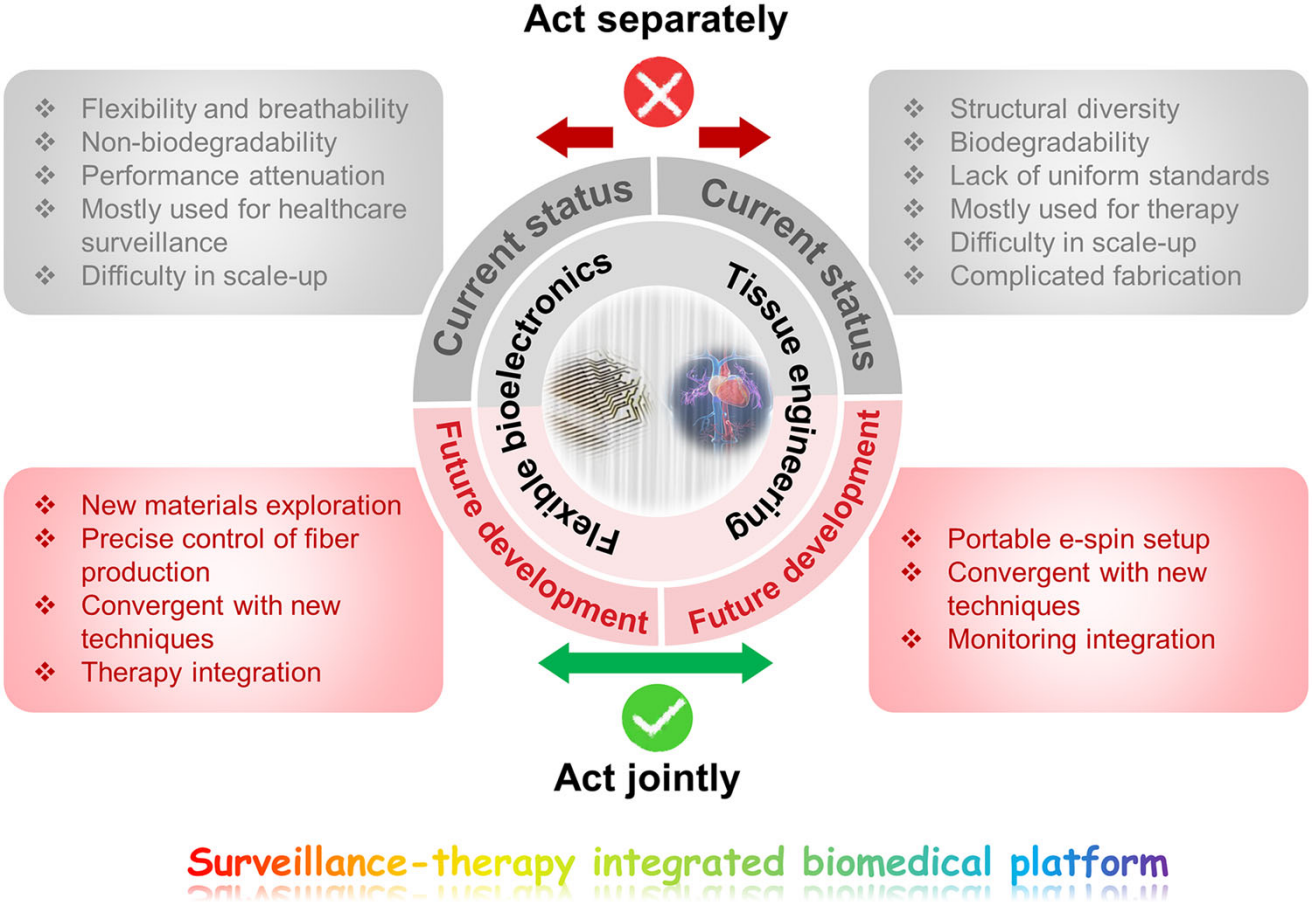
Summative scheme of the current status and future development of e‐spin fiber‐based materials in the application of flexible bioelectronics and tissue engineering

In summary, great progress has been made on the development of e‐spin technology for numerous applications, among which flexible bioelectronics and tissue engineering are the most cutting‐edge and intriguing ones. Micro/nanofiber‐based materials and devices with hierarchical structures and biomimetic architectures from have been successfully fabricated via designing the e‐spin apparatus and optimizing processing factors. For flexible bioelectronics and tissue engineering, the high compatibility, bionic structure, cell induction ability, controllable degradability, and proper mechanical property should be considered when designing the e‐spin scaffolds and devices. With the integration of different components including organic polymers, inorganic nanoparticles, biomolecules, imaging agents, therapeutic drugs, living cells, bacteria, and vaccines during the e‐spin procedure, plenty of functional electrospun materials have been reported. These e‐spin or e‐spin‐derived materials and devices show broad applications in the field of flexible bioelectronics and tissue engineering for the purpose of biosensing, monitoring, therapy, and intelligent healthcare. These amazing developments encourage the researchers to go on through thorns. During the course, the existing challenges and future perspectives should be recognized, especially toward mature industrialization and clinical transformation (Figure 6).

### 4.1 Optimization of the e‐spin apparatus and technology

The stability, repeatability and high efficiency of e‐spin technology are the preconditions for further application of the e‐spin fibers. The mass production ability of e‐spin is anticipated for large‐scale commercialization. Among the different applications, flexible electronics especially wearable electronics are near close to real applications.

For improving the technology and fabrication equipment, multiple‐needle, and needleless e‐spin hold great promise in expanding fiber production and have been used as designation rationale for industrial manufacture. There is a commercial apparatus that integrates 110 needles in the e‐spin apparatus for mass production.^[^ ^70^ ^]^ Despite many efforts still remain in the laboratory stage, more precise regulation of e‐spin fiber deposition and formation have also been achieved by the integration with other technologies such as, microfluidics,^[^ ^71^ ^]^ melt electrowriting,^[^ ^72^ ^]^ and electrospraying.^[^ ^73^ ^]^ Meanwhile, the bulky instrument of conventional e‐spin limits the realization of popularization and clinical applications. To this end, advanced portable e‐spin devices are explored in the form of hand‐held spinnerets, battery‐, and generator‐powered devices.^[^ ^74^ ^]^ These developing e‐spin devices can realize low‐cost, in situ and precise fiber deposition on the target site (typically wound bed) without utilization of high‐voltage system. This may lead a technical revolution for next‐generation intelligent and personalized healthcare.

### 4.2 Extension of materials for e‐spin

On the premise of the universality of e‐spin for most polymers, the exploitation of materials suitable for e‐spin for targeted applications is still required for maximizing the integrated performance.

For instance, newest researches found that PVDF‐based piezo‐polymers had higher ferroelectricity after electrospinning stretching and electrical polarization. Filler incorporation during e‐spin can also reinforce the PVDF chains to rearrange into the piezoelectric β phase with for optimal mechano‐electro conversion.^[^ ^34^, ^75^ ^]^ By tuning the calcination conditions, flexible piezoelectric biosensors composed of inelastic inorganic ceramics such as, BaTiO_3_ can be obtained and large‐scale fabricated,^[^ ^76^ ^]^ which can overcome the low piezoelectricity of traditional flexible piezoelectric polymers. As a result, it can accelerate the progress on wearable and implantable bioelectronics for detecting weak physiological signals.

Hydrogels are another class of materials being studied for electrospinning. Its innate property of high‐water content and tunable mechanical properties can be customized to fit the specific tissue, thus promoting the progress on flexible sensing and regenerative medicine.^[^ ^77^ ^]^ E‐spin technology can assist in the directional fibrous structure of hydrogel, which is difficult to be realized by other methods. As above discussed, this kind of structure benefits the alignment of electroactive cells to promote signal transmission.^[^ ^78^ ^]^ Moreover, e‐spin fibers with “smart” properties such as reversibly stimuli‐responsive, shape memory, self‐sacrificing, self‐cleaning, and self‐healing properties can also reinforce the corresponding performance and deepen the existing research contents.^[^ ^1^, ^79^ ^]^

### 4.3 Exploration of multifunctional devices based on e‐spin fiber

Currently, the application of e‐spin fabric is widespread over many fields. Intelligent fabric with multiple functions is also under developing to meet the practical needs of the Internet of Things. Through appropriate micromachining technology, the combination of bioelectronics with regenerative medicine in a two‐in‐one manner is expected to realize intelligent sensing and therapy. By integrating the implantable sensor into an e‐spin fibrous interface, both biophysical and biochemical signals can be directly delivered to cells or tissues. And the signals generated under the tissue reconstruction during healing process can be given back through sensor module in real time to form a self‐feedback theranostic system.^[^ ^80^ ^]^ For example, in the treatment of cardiac diseases, e‐spin based devices can not only serve as a tissue scaffolds to regulate the beating rhythm of cardiomyocytes but also deliver drugs and electrostimulation to cells on demand. Corresponding cellular electrical activities under external stimulation can also be recorded.^[^ ^40^ ^]^ This multifunctional device integrated energy harvesting module, controllable drug delivery system, and flexible sensors, thus realizing timely monitoring of the treatment process. In addition to multifunctional integration, another major task is to solve the poor transmission of implanted device through biological tissues. It may be achieved through integrating wireless communication in one autonomous device without any external interference. For further development, full considerations should be taken on the in vivo safety issues involving biocompatibility, controllable biodegradability, long‐term function stability, immunoregulatory concern,^[^ ^81^ ^]^ and adaptability to dynamic mechanical environment.^[^ ^82^ ^]^ With all these efforts, it is expected that the e‐spin technology, materials, and devices will revolutionarily promote the development of personalized medicine and intelligent healthcare.

### 4.4 Other considerations

In addition to the above‐mentioned aspects, other considerations for the development of electrospun material should be taken. Uniformity is the primary preconditions to maintain long‐term excellent performance no matter for biological applications or the stability of flexible electronics. Batch preparation can fasten the parameter optimization for e‐spin‐based materials. By integrating the 3D printing with e‐spin technique, the fibrous materials with precise spatial and temporal control will be obtained easily, which is meaningful to decrease lead time and cost for accelerating the basic research toward transformation.^[^ ^83^ ^]^ In addition, the acquisition of standardized e‐spin product for scale‐up also requires the joint efforts from multiple disciplines. Systematical theoretical modeling and calculation can become an auxiliary mean to assist in totally repeated designing specific nanofibers for different application demands.

## CONFLICT OF INTEREST

The authors declare no conflict of interest.
